# Collaborative research between academia and industry using a large clinical trial database: a case study in Alzheimer's disease

**DOI:** 10.1186/1745-6215-12-233

**Published:** 2011-10-26

**Authors:** Roy Jones, David Wilkinson, Oscar L Lopez, Jeffrey Cummings, Gunhild Waldemar, Richard Zhang, Joan Mackell, Serge Gauthier

**Affiliations:** 1 RICE (Research Institute for the Care of Older People), Royal United Hospital, Combe Park, Bath BA1 3NG, UK; 2 Memory Assessment and Research Centre, Moorgreen Hospital, Southampton, UK; 3 Alzheimer's Disease Research Center, Departments of Neurology and Psychiatry, University of Pittsburgh, Pittsburgh, PA, USA; 4 Cleveland Clinic, Lou Ruvo Center for Brain Health, Las Vegas, NV, USA; 5 Memory Disorders Research Group, Dept. of Neurology, Copenhagen University Hospital, Rigshospitalet, Copenhagen, Denmark; 6 Pfizer Global Pharmaceuticals, Pfizer Inc, New York, NY, USA; 7 McGill Center for Studies in Aging, Douglas Mental Health University Institute, Douglas Hospital, Verdun, Quebec, Canada

## Abstract

**Background:**

Large clinical trials databases, developed over the course of a comprehensive clinical trial programme, represent an invaluable resource for clinical researchers. Data mining projects sponsored by industry that use these databases, however, are often not viewed favourably in the academic medical community because of concerns that commercial, rather than scientific, goals are the primary purpose of such endeavours. Thus, there are few examples of sustained collaboration between leading academic clinical researchers and industry professionals in a large-scale data mining project. We present here a successful example of this type of collaboration in the field of dementia.

**Methods:**

The Donepezil Data Repository comprised 18 randomised, controlled trials conducted between 1991 and 2005. The project team at Pfizer determined that the data mining process should be guided by a diverse group of leading Alzheimer's disease clinical researchers called the "Expert Working Group." After development of a list of potential faculty members, invitations were extended and a group of seven members was assembled. The Working Group met regularly with Eisai/Pfizer clinicians and statisticians to discuss the data, identify issues that were currently of interest in the academic and clinical communities that might lend themselves to investigation using these data, and note gaps in understanding or knowledge of Alzheimer's disease that these data could address. Leadership was provided by the Pfizer Clinical Development team leader; Working Group members rotated responsibility for being lead and co-lead for each investigation and resultant publication.

**Results:**

Six manuscripts, each published in a leading subspecialty journal, resulted from the group's work. Another project resulted in poster presentations at international congresses and two were cancelled due to resource constraints.

**Conclusions:**

The experience represents a particular approach to optimising the value of data mining of large clinical trial databases for the combined purpose of furthering clinical research and improving patient care. Fruitful collaboration between industry and academia was fostered while the donepezil data repository was used to advance clinical and scientific knowledge. The Expert Working Group approach warrants consideration as a blueprint for conducting similar research ventures in the future.

## Background

Development of any therapeutic agent requires investment in a comprehensive clinical trial programme. Over the developmental lifespan of the drug, many large and small studies are undertaken. All the accumulated data are securely stored in databases. These databases represent a huge potential resource. In other industries--ranging from manufacturing to marketing, travel to banking, and telecommunications to finance--data mining, the nontrivial extraction of implicit, previously unknown patterns and relationships from data, is a highly valued enterprise [1]. Independent, not-for-profit organisations such as the Cochrane Collaborative http://www.cochrane.org, and governmental bodies, such as the Agency for Healthcare Research and Quality http://www.ahrq.gov/research, provide excellent examples of thoughtfully constructed and expertly executed data mining research of clinical trial data.

There are, however, few examples of attempted or successfully sustained collaboration between leading academic clinical researchers and industry professionals in such an effort. To the best of our knowledge, we present here the first example of large-scale, extended collaboration of this type in the field of dementia. This article describes how one drug development team, in partnership with key therapy area experts, used their database of clinical trials of donepezil, a symptomatic treatment for patients with Alzheimer's disease (AD), to address questions beyond the scope of the original studies. This collaboration, titled the "Donepezil Expert Working Group project," resulted in numerous publications addressing important clinical and scientific questions concerning AD and its treatment.

## Methods

### Background

Donepezil--E2020; (R, S)-I-benzyl-4[(5,6-dimethoxyl-indanon)-2-yl]-methyl piperidine hydrochloride--is a piperidine-based derivative, chemically distinct from the other cholinesterase inhibitors (ChEIs). Developed specifically for the treatment of AD, donepezil is a highly selective, reversible, noncompetitive inhibitor of acetylcholinesterase (AChE). Donepezil, marketed under the trade name Aricept^® ^by its developer Eisai and partner Pfizer, received approval from the US Food and Drug Administration (FDA) for treatment of mild to moderate AD in November 1996 and is now approved in over 50 countries for this indication. The indication was expanded to include severe AD in October 2006 in the United States and in August 2007 in Japan.

### The Donepezil AD Database

The donepezil data repository comprises primarily US Pfizer and US Eisai clinical trial data from phase 2, phase 3 and phase 4 trials. In total, the database contains 27 integrated AD studies (23 in patients with mild/moderate AD, one in patients with moderate to severe AD and three in patients with severe AD), including 18 double-blind, placebo-controlled trials, four open-label extension studies, two clinical experience trials, and three others. Data for more than 6000 unique patients, treated with donepezil for up to 5 years, are contained in the repository [2-7].

Table 1 displays summary information for all 18 randomised, controlled trials, which comprised the data base for the data mining project.

**Table 1:** Donepezil AD data repository summary (randomised, controlled trials only)

Study identifier	Phase	Disease stage/MMSE range	Donepezil/placebo dose, mg	Duration (weeks)	Study objective for donepezil	Total patient number (donepezil + placebo)	Cognitive efficacy measure	Period of study	EWG study*
201 [8]	2	Mild to moderate/10-26	1, 3, 5	12	Initial study of efficacy and safety	150	ADAS-cog MMSE	1991-1992	1, 3, 5

301 [9]	3	Mild to moderate/10-26	5, 10	12	Pivotal study of efficacy and safety (USA)	476	ADAS-cog MMSE	1993-1994	1, 5

302 [9]	3	Mild to moderate/10-26	5, 10	24	Pivotal study of efficacy and safety (USA)	471	ADAS-cogMMSE	1993-1994	1, 2, 5

304 [10]	3	Mild to moderate/10-26	5, 10	24	Pivotal study of efficacy and safety (Europe)	816	ADAS-cog	1994-1996	1, 3, 5

134 [11]	2	Mild to moderate/10-26	3, 5	12	Efficacy and safety (Japan)	190	ADAS-cog	1994	5

161 [12]	3	Mild to moderate/10-26	5	24	Efficacy and safety (Japan)	268	ADAS-cog	1996-1999	1, 5

203 [13]	2	Mild to moderate/10-26	10	24	Effects on regional brain glucose metabolism	28	ADAS-cog	1996-1997	1, 5

204 [14]	2	Mild to moderate/10-26	10	24	Effects on hippocampal volume and neuron integrity	67	ADAS-cog	1996-1997	1, 5

205 [15]	2	Mild to moderate/10-26	10	52	Effects on visuospatial awareness	11	MMSE	1996-1997	1, 5

306 [11]	3b		10	12	ApoE subtype and response	38	MMSE	1997-1998	1, 5

311 [16]	3b	Mild to severe/5-26	10	24	Efficacy and safety in nursing home patients	208	MMSE	1996-1997	1, 3, 5

312 [17]	3b	Mild to moderate/12-20	10	54	Preservation of function	431	MMSE	1996-1997	1, 3, 5

324 [18]	3b	Moderate to severe/5-18	10	24	Efficacy and safety in moderate to severe AD	290	MMSE, SIB	1996-1998	1, 2, 3, 4, 5, 6

33301	3b	Mild to moderate/18-26	10	12	Effect on attention	296		1997	

96001 [19]	3b	Mild to moderate/10-26	10	52	1-year DB efficacy and safety	286	MMSE	1997	1, 2, 3, 5, 6

231 [20]	4	Severe/1-12	10	24	Efficacy and safety in severe AD	343	SIB	2001-2005	4

315 [21]	4	Severe/1-12	5, 10	24	Efficacy and safety in severe AD (Japan)	248	SIB	2002-2005	4

1017 [22]	4	Severe/1-10	10	24	Efficacy and safety in severe AD	302	SIB	2002-2004	4

*1 = Jones RW et al [2]; 2 = Wilkinson D et al [3]; 3 = Gauthier S et al [4]; 4 = Cummings J et al [5]; 5 = Lopez O et al [6]; 6 = Waldemar G et al [7].

MMSE = Mini-Mental State Examination, EWG = Expert Working Group, ADAS-cog = Alzheimer's Disease Assessment Scale-cognitive subscale, ApoE = apolipoprotein E, SIB = Severe Impairment Battery, AD = Alzheimer's disease, DB = double blind.

### The Expert Working Group

The project was initiated by Pfizer donepezil clinical teams. The rationale for the project was to take advantage of the potential of the AD database to address important disease- and treatment-related questions that individual trials do not. The main objectives of the project were to identify a set of clinical questions that could be addressed by data mining, to prioritise the potential projects and determine which ones to pursue and to carry out these projects with the intention of presenting the findings at major congresses and publishing them in leading peer-reviewed academic journals.

The donepezil team at Pfizer agreed that the data mining process should be guided throughout by a diverse group of leading AD clinical researchers, which was termed the "Expert Working Group" or EWG. Experts were chosen because of their knowledge of the therapeutic area, familiarity with clinical trial methodology and outcome measurement, participation in some of the trials sponsored by Pfizer and/or Eisai and willingness to participate actively in the EWG programme. After a list of potential EWG faculty members was developed, invitations were extended and a group assembled. The group was specifically chosen to represent the global nature of the AD clinical trial programme, and was small enough to facilitate optimal group cohesion and functioning.

The EWG was officially established in September 2005 at a meeting in Rome, Italy; the five initial faculty members of the EWG were Jeffrey Cummings (JC), Howard Feldman (HF) (who left in 2008 after taking an industry position), Roy Jones (RJ), Gunhild Waldemar (GW) and David Wilkinson (DW). Based on questions the group developed at this inaugural meeting, the Chair (RJ) and faculty members recommended two additional participants, Serge Gauthier (SG) and Oscar Lopez (OL), who joined subsequently. Geographically, the experts were based in the United Kingdom (RJ, DW), Denmark (GW), Canada (SG, HF), and the United States (JC, OL). Then, as now, all were professors, clinicians and researchers in the field of AD and dementia. Research interests of group members included clinical trials of drugs for AD and other dementias, immunotherapy for AD, neuropsychiatric and behavioural symptoms in AD, early and prodromal AD, brain structure and imaging, and genetics. Collectively, they had published more than 1000 papers on dementia and related issues.

The core goal of the EWG was to investigate aspects of AD or its treatment that were insufficiently addressed by the single studies but that could potentially be addressed using the clinical trial database. In order to accomplish this, Pfizer agreed to provide unfettered access to the data repository for the EWG members. In practice, this meant that relevant data tables and summaries were provided upon request with no restrictions (EWG members did not actually have direct access to the data repository, primarily due to its unwieldy size and idiosyncratic organisation). The EWG members met regularly to discuss the data, identify gaps in understanding or knowledge of AD that were currently of interest in the academic and clinical communities that might lend themselves to investigation using these data and suggest novel or innovative analyses that could potentially address these questions. Seven Eisai/Pfizer clinicians and three statisticians participated in the EWG project over its 4.5-year duration. In addition, a medical communications agency provided logistical and editorial support for EWG meetings, congress presentations and manuscript development.

### EWG Activities

At the first EWG meeting, the group discussed numerous investigations that could be carried out using the data repository for the following three general categories: (1) the nature of AD; (2) treatment with donepezil; and (3) clinical trial methodology. In total, eight analyses were identified and prioritised by the EWG as worthy of study. Two of these analyses with the lowest level of interest were not carried out because of resource constraints. As each research question was identified, various analyses were suggested and evaluated.

For each project, a lead and co-lead academic clinical researcher were identified. Although all EWG members participated in all projects, it was important to assign primary responsibility for each of the individual projects to specific members. A detailed statistical analysis plan was drafted, distributed to the EWG members and discussed via teleconference and email, resulting in a finalised set of analyses to be undertaken. Results of all analyses, including generated data tables and figures, were shared with all group members; decisions regarding which investigations to pursue further were taken by consensus of the EWG group. Subsequent publication development was also determined by EWG members. Authorship was determined by level of individual interest and participation as well, so not all EWG members were authors on every manuscript.

Over the course of 4 years, the group met six times (Rome, Italy, November 2005; Glasgow, UK, September 2006; London, UK, November 2007; Hong Kong, China, February 2008; Chicago, IL, USA, July 2008; New York, NY, USA, via WebEx, April 2009), often in conjunction with a major international AD meeting. Leadership was provided by the Clinical Development team leader, supported by the scientific lead at the medical communications agency. Members of the group rotated responsibility for being lead and co-lead for each of the separate investigations and resultant publications. All members of the EWG received remuneration (honoraria) from Pfizer Inc for their participation in the EWG meetings, but not for time devoted to manuscript development.

## Results

Six published manuscripts resulted from the work of the EWG. Each manuscript focused on a different topic and had its own unique selection criteria for included studies (Table 2).

**Table 2:** EWG project summary

Project title	RCTs included (n)	Selection criteria	n			Citation
				
			Total	Donepezil	Placebo	
Rates of cognitive change in AD: observations across a decade of placebo-controlled clinical trials with donepezil	13	Baseline MMSE scores between 10 and 26 Received placebo	3403	NA	3403	[2]

Effectiveness of donepezil in reducing clinical worsening in patients with mild to moderate AD	3	Baseline MMSE scores between 10 and 26 Duration of at least 24 weeks Available patient-level data for cognitive, global and functional assessments	906	518	388	[3]

Predicting cognitive decline in AD: an integrated analysis	14	Baseline and end-of-study (12- or 24-week) data on cognition, (MMSE and/or ADAS-cog)	3748	2238	1510	[6]

Effect of donepezil on emergence of apathy in mild to moderate AD	2	MMSE scores between 10 and 26 At least 24 weeks' treatment duration Individual patient-level behavioural data using the NPI available	490	241	249	[7]

Effects of donepezil on activities of daily living: integrated analysis of patient data from studies in mild, moderate and severe AD	6	Available post-baseline functional data collected using at least 1 ADL scale as defined in the original study protocols	2194	1268	926	[4]

Effect of donepezil on cognition in severe AD: a pooled data analysis	4	Available SIB data at both baseline and at study end	904	481	423	[5]

RCTs = randomised, controlled trials, AD = Alzheimer's disease, MMSE = Mini-Mental State Examination, ADAS-cog = Alzheimer's Disease Assessment Scale-cognitive subscale, NPI = Neuropsychiatric Inventory, ADL = activity of daily living, SIB = Severe Impairment Battery.

### Understanding changes in placebo populations over time

The database was used to compare the rates of cognitive decline as measured by the Mini-Mental State Examination (MMSE) or the Alzheimer's Disease Assessment Scale-cognitive subscale (ADAS-cog) in placebo-treated patients enrolled in trials with start dates between 1990 and 1994 (Group 1) versus those enrolled in trials with start dates between 1996 and 1999 (Group 2) (Table 1) [2].

The major finding was that at 24 weeks, placebo-treated patients from Group 2 (those enrolled in trials beginning after 1995) had a significantly slower rate of decline than those in Group 1 (participants in trials beginning prior to 1995). Thus, over the 10 years of the donepezil clinical trial programme, placebo groups, which were expected to remain similar, showed slower rates of cognitive decline in more recent trials compared with older trials. This finding suggests that the start date of AD clinical trials may need to be considered as an important variable when comparing study results and that the duration of future trials may need to be extended to account for the slower rate of cognitive decline in the placebo group.

### Reconceptualising treatment effect as a reduction in clinical decline

AD is a disease of unremitting deterioration in cognition and the ability to perform the tasks of everyday living. Therefore, it is important to recognise that a reduction in the rate of decline is a positive treatment outcome, even if no improvement relative to baseline occurs. In addition, identifying clinical characteristics associated with more rapid decline may be helpful in teasing out treatment effects. Thus, two studies were conceived to explore the nature of decline in patients with AD and the effect of donepezil treatment on clinical decline.

The first examined whether patients receiving donepezil treatment had "reduced worsening" (operationally defined by group consensus) compared with placebo [3]. Data on cognition, global change and function were available from all patients at baseline and end point for the three studies used in this analysis (Table 1). Clinical worsening at Week 24 of the studies was assessed using three sets of criteria: decline in cognition only (COG); decline in cognition plus global rating (COG + G); and decline in cognition, global rating and functional assessment (COG + G + F).

The main finding of the analyses was that significantly more placebo patients met the prespecified criteria for all three definitions of clinical worsening (COG, COG + G, COG + G + F) than donepezil-treated patients, including after stratification by baseline severity. This analysis demonstrated the impact of treatment in reducing decline in cognition, global ratings and function (activities of daily living; ADL), and provides support for this alternative measure of treatment effectiveness. This has implications for future study design and for the assessment of treatment success.

In the second study focusing on the theme of decline, in which 14 of 18 studies were included (Table 1), the goal was to identify baseline demographic or illness characteristics associated with more rapid cognitive decline and to determine whether donepezil treatment had an impact on the rate of decline in the subgroup of patients with rapid decline [6]. Factors shown previously to influence cognitive deterioration were assessed for their ability to predict more rapid cognitive decline. Patients were divided into "fast decliners" and "slow decliners" according to two criteria. Criterion 1 was based on reported average annual rates of cognitive decline in untreated AD patients; Criterion 2 utilised the pooled placebo population from the included studies, classifying patients into three equally sized groups (tertiles) according to their observed change in MMSE or ADAS-cog scores over the 12- or 24-week trials.

The key findings were as follows: variables that were significantly associated with fast cognitive decline were younger age, poorer cognitive and/or behavioural status at baseline and absence of diabetes. Treatment with donepezil reduced the odds of a fast decline in MMSE by 39% to 52% and in ADAS-cog by 57% to 63%. These results provided supportive evidence with respect to some factors previously associated in the literature with faster cognitive decline. They also demonstrated treatment effects in terms of reducing the rate of decline.

### Exploring AD symptom domains

The EWG recognised "apathy" as one of the early and often persistent cardinal symptoms of the disease, a symptom that caregivers rate as reducing the quality of life of the patient with AD [23]. The EWG was interested in understanding the natural history and treatment response of this symptom in comparison to other behavioural symptoms characteristic of AD [7]. To increase clinical relevance, a milestone (categorical) approach was used, rather than comparing group means on a continuous measure. A clinical behavioural milestone was defined as the first emergence post baseline of a Neuropsychiatric Inventory (NPI) item composite score (frequency × severity) ≥ 3 for that behaviour. Differences in time to milestone for apathy and other NPI-rated behaviours were assessed in both placebo- and donepezil-treated groups. Just two studies were included because the NPI was not widely utilised in donepezil clinical trials (Table 1).

Apathy was the NPI item with the highest proportion of patients having a composite score ≥ 3 at baseline, roughly 50%. Time-to-event analysis demonstrated a significant delay in reaching the clinical milestone for apathy in patients treated with donepezil compared with those receiving placebo (*P *= 0.01). A significant delay was also noted for aberrant motor activity (*P *= 0.04); results for the other NPI items were not significant. This analysis confirmed prior reports of the clinical course of apathy in AD [24] and provided preliminary evidence that ChEI treatment may delay its onset or exacerbation. In addition, the analysis found that depression (as an NPI item) showed a markedly different pattern than apathy, suggesting that apathy is not primarily an epiphenomenon of depression.

### Standardising various functional scales

In AD clinical research, as well as that of numerous other chronic conditions, a variety of rating scales are used to measure daily functioning. Consequently, results are often not comparable across studies. In an ideal world, one test scale would be used consistently across trials. One way to develop such a "standardised functional scale" is to systematically integrate elements of existing scales. The EWG sought to employ the donepezil clinical trials database to pursue this goal. Secondarily, pooling functional outcome data was used to better characterise the details of functional decline in AD and the impact of treatment [4].

For this analysis, six randomised, double-blind placebo-controlled studies of 12 (one study) or 24 weeks' duration were selected based on availability of post-baseline functional data (Table 1). Individual items from the nine ADL scales used in these studies were mapped to a standardised functional scale comprising 12 domains--six basic and six instrumental. Individual items from each scale were mapped to the new standardised scale by the EWG statistician (RZ) and scores were transformed to a 0-100 scale. External validation of item mapping by a recognised expert in the field of functional assessment yielded a concordance rate of 90.8%, suggesting that the process was highly reliable. Patient data from the six original trials (2177 patients with MMSE scores between 5 and 26) were then scored using the new standardised scale.

Donepezil treatment was associated with significantly less worsening than placebo on change from baseline to end of study for five items of the standardised scale (*P *< 0.05), and numerically less worsening on 11 of the 12 standardised items. Dividing the patients into subpopulations with mild (MMSE 18-26), moderate (MMSE 10-17) and severe (MMSE 5-9) AD showed that treatment with donepezil provided the most functional benefit to those in the moderate phase of the disease.

This work provided an example of a method to standardise multiple functional scale scores, allowing for comparison of functional outcomes between studies employing different rating scales and/or pooling of functional data from multiple studies to increase statistical power. It also provided additional supportive evidence for the benefit of ChEI treatment on daily functioning, particularly in patients with AD of moderate severity.

### Investigating the utility of treatment in patients with severe AD

The final EWG project focused on the controversial question of the utility of treating patients with severe AD with ChEIs. Licensing of ChEIs for severe AD remains inconsistent across countries, and the clinical meaningfulness of the drugs' use in this population has been questioned [25]. The EWG believed that part of this inconsistency was a consequence of the poor performance of standard instruments, such as the MMSE or ADAS-cog, in capturing the natural history of the disease and the effects of treatment in this patient population. For this reason, studies using the Severe Impairment Battery (SIB), which has been shown to track change over time in patients with late-stage AD [26], were selected (Table 1). These analyses sought to better explain the performance of the SIB in clinical trials and to examine in detail the impact of treatment with donepezil in this population [5]. For the analysis, patients were stratified by baseline MMSE scores into four groups: MMSE score 1-5 (most severe), MMSE score 6-9 (severe), MMSE score 10-12 (early severe) and MMSE score 13-17 (moderate).

The main finding of this study was a significant least squares (LS) mean difference in SIB total scores from baseline to 24 weeks between those treated with donepezil and those receiving placebo (6.22 [*P *< 0.0001, Cohen's *d*, 0.53]). Treatment-placebo LS mean differences were statistically significant for all baseline severity strata (range, *P *= 0.0251 to *P *< 0.0001; Cohen's *d*, 0.41-0.66) and for seven of nine SIB domains (range, *P *= 0.0056 to *P *< 0.0001; Cohen's *d*, 0.17-0.48). Patients receiving donepezil showed improvement above baseline in eight of nine domains, those receiving placebo worsened in all domains. The clinical meaningfulness of these improvements was demonstrated with significant positive correlations between change in SIB scores and change in functional and global measures.

## Discussion

In this article we seek to share, in broad outline, the organisational process used by the donepezil EWG for identifying scientific questions of interest and carrying out exploratory research using a large clinical trial database. We then illustrate the value of this approach for elucidating useful information about AD and the impact of donepezil treatment by summarising the EWG's publications output. Through this process, academic and clinical researchers gained access to a vast database, important questions concerning AD and its treatment were addressed, and industry funding was used to support research beyond narrow regulatory goals.

There were numerous clinically relevant findings from the EWG studies, including extending knowledge of placebo group outcomes in AD clinical trials by documenting a trend towards slower decline from 1990 to 2000; illustrating the value of using "reduced worsening" (or "less than expected decline") as an outcome measure of treatment effectiveness in AD studies; providing evidence that the onset/exacerbation of apathy can be delayed by AChEI treatment; demonstrating a method for standardisation of multiple functional scales, enabling improved comparisons across studies and pooling of functional data from multiple studies; and using the SIB to document improvement in patients with severe AD. Each paper either provided supportive evidence for existing hypotheses concerning disease progression and/or response to treatment or was regarded as hypothesis generating and helped to build the case for additional prospective research on new topics.

The size of the database was a great asset for these studies, as data could be divided into groups that all retained sufficient numbers of patients for meaningful statistical comparisons. For example, in the severe AD manuscript [5], patients with severe AD were subdivided into three groups according to their MMSE score; in the manuscript investigating rapid decline [6], patients were stratified into fast and slow decliners using two different sets of criteria. The extended span of time covered by the database was critical in being able to detect a change in the performance of placebo groups from 1990 to 2000.

There were several key strengths of the EWG approach that differentiate it from routine pharmaceutical industry data mining research. Most importantly, the investigations were suggested, evaluated and led by leading clinical researchers in the field of AD and dementia, not by the company. Although this was a partnership between professionals in industry and academia, it was the former who provided the resources while the latter guided the process. Second, the company's data were extensively shared with EWG members. Third, the medical communications agency was treated as a true partner in the collaboration, which enhanced the organisational effectiveness and publications productivity of the group as a whole.

Despite the strengths of the project, there are inherent limitations to all data pooling research that also apply to the work of the EWG. For example, large databases can sometimes assist in identifying statistically significant outcomes that are not clinically meaningful. The EWG was cognisant of this threat and used a variety of approaches to confirm the clinical importance of the results. Also, by definition, all of the analyses were post-hoc and therefore exploratory. In addition, all involved pooling of data from trials that were conducted according to different protocols, in different places, and at different times. The repository data were limited to company-sponsored donepezil trials, so studies on comparative effectiveness were not possible and investigator-initiated trials in other populations were not included. Finally, there is always a potential bias based on the particular experts selected and the topics chosen (and not chosen) for analysis. However, the size and diversity of the group were intended to minimise this source of bias.

There are certain aspects of the EWG process that could be improved in future projects of this type. First, the delay between completion of clinical trials and initiation of the data mining effort could be shortened. For example, a project similar to the EWG could be initiated prior to the completion of the entire clinical trial programme, particularly if subsequent clinical trials focus on a different stage of the disease, or a different manner of therapy (e.g. add-on vs. monotherapy), than the component of the trial programme already completed. With respect to the EWG project, during the 5 years between completion of the randomised, controlled trials in mild to moderate AD and the first meeting of the EWG, three large clinical trials in severe AD and a number of open-label studies were all being conducted, which limited the capacity of the company to begin a major new project. Second, although the final EWG meeting did take place via Web conferencing, this technology is continually improving and could be utilised more extensively in addition to, as well as in place of, live meetings.

Use of clinical trial databases is becoming more important because of concerns about patient safety and the initiative to study comparative effectiveness of treatments. As such, it will require cooperation among pharmaceutical companies, and between pharmaceutical companies and regulatory agencies, particularly the US FDA. In this regard, the FDA has launched a new project, called Janus, which is currently gaining momentum. Janus will provide a hub for integrating data within the FDA to support regulatory decisions and pharmacovigilance. The goal is to facilitate standardisation in acquisition and analysis of study data, allowing integration of clinical trial data and postmarketing safety data to improve public health and patient safety. The Janus repository was created during 2010, and existing data are being converted into the standard format during 2011. This project will harness the capacity of large study data repositories to answer questions about care and to enable pilot studies of comparative effectiveness [27]. Two other examples of effective data sharing are the Critical Path Institute's Coalition Against Major Diseases database of negative AD trials (C-Path Online Data Repository, http://www.c-path.org) and the AD Neuroimaging Initiative (ADNI, http://www.adni-info.org). In this regard, the EWG process is a stepping stone towards much deeper and wider degrees of partnership and collaboration in the service of patient safety and treatment effectiveness.

## Conclusion

The EWG experience represents a particular approach to optimising the value of data mining of large clinical trial databases for the combined purpose of furthering clinical research and improving patient care. Fruitful collaboration between industry and academia was fostered while the donepezil data repository was used to advance clinical and scientific knowledge. The EWG approach warrants consideration as a blueprint for conducting similar research ventures in the future.

## Competing interests

The analyses described herein result from a constituted expert working group initiated and funded by Eisai Inc. and Pfizer Inc. Drs Richard Zhang, Yikang Xu and Joan Mackell are employees of Pfizer Inc. All other authors report having received consultancy and/or speaker fees from Pfizer Inc and each author also received honoraria for their participation in the expert working group sessions; none received any compensation for their work on this manuscript. Dr. Cummings holds the copyright of the Neuropsychiatric Inventory.

## Authors' contributions

All authors contributed equally to this manuscript, and to the analyses and publications described in the manuscript. Each author participated in the concept and design of this article, reviewed each draft and provided critical intellectual content and approved the final manuscript.
